# Passivation Mechanism of (18-Crown-6) Potassium on Complex Defects in SnO_2_ Electron Transport Layer of Solar Cells

**DOI:** 10.3390/molecules30204081

**Published:** 2025-10-14

**Authors:** Shiyan Yang, Qiuli Zhang, Qiaogang Song, Yu Zhuang, Shurong Wang, Youbo Dou, Jianjun Wang, Xintong Zhao, Longxian Zhang, Hongwen Zhang, Wenjing Lu, Xihua Zhang, Yuan Wu, Xianfeng Jiang

**Affiliations:** 1 School of Energy and Environment Science, Yunnan Normal University, Kunming 650500, China; 18184845393@163.com (S.Y.); 15087540821@163.com (Q.Z.); shrw88@aliyun.com (S.W.); ybdou@outlook.com (Y.D.); wjianjun622@gmail.com (J.W.); xtzhao21@163.com (X.Z.); 2380160027@ynnu.edu.cn (L.Z.); 18287442863@163.com (H.Z.); 15887460619@163.com (W.L.); 15911967478@163.com (X.Z.); 15198713255@163.com (Y.W.); 17761402464@163.com (X.J.); 2 Key Laboratory of Novel Photovoltaic Materials and Thin-Film Solar Cells of Yunnan Provincial Department of Education, Yunnan Normal University, Kunming 650500, China

**Keywords:** (18-crown-6) potassium, SnO_2_, defect passivation, first-principles calculations

## Abstract

In this study, first-principles calculations were employed to systematically investigate the interaction mechanisms between (18-crown-6) potassium (18C6-K^+^) and six typical defect sites on the SnO_2_ (110) surface, including Sn_i_ + Sn_O_, O_i_ + O_Sn_, V_O_ + Sn_i_, V_Sn_ + Sn_O_, V_Sn_ + Sn_i_, and Sn_i_. Six intrinsic or complex defects universally coexist on the SnO_2_ surface, and the defect states they introduced allow for precise tuning of material performance. The results demonstrated that the 18C6-K^+^ molecule can stably adsorb on all six defect sites and significantly increase defect formation energies, indicating its thermodynamic capability to suppress defect generation. A subsequent density of states (DOS) analysis revealed that the 18C6-K^+^ molecule exhibits strong defect passivation effects at Sn_i_ + Sn_O_, V_O_ + Sn_i_, V_Sn_ + Sn_i_, and Sn_i_ sites, and partially mitigated the electronic disturbances induced by O_i_ + O_Sn_ and V_Sn_ + Sn_O_ defects. Furthermore, the incorporation of 18C6-K^+^ has been shown to reduce the electronic effective mass of defective systems, thereby enhancing surface carrier transport. A subsequent charge density difference (CDD) analysis revealed that the 18C6-K^+^ molecule forms Sn-ether and O-ether interactions through its ether bonds (C-O-C) with surface Sn and O atoms, inducing interfacial electronic reconstruction and charge transfer. The Bader charge analysis revealed that the H, C, and O atoms in 18C6-K^+^ lose electrons, whereas the Sn or O atoms at the surface defect sites gain electrons. This outcome is consistent with the CDD analysis and quantitatively confirms the extent of electron transfer from 18C6-K^+^ to the SnO_2_ defect regions. These interactions effectively passivate defect states, thereby enhancing interfacial stability. The present study offers theoretical guidance and design insights for the development of molecular passivation strategies in SnO_2_-based optoelectronic devices.

## 1. Introduction

Tin oxide (SnO_2_), a wide-bandgap semiconductor (∼3.6 eV) [1], has found extensive applications in solar cells, transparent conductive oxide (TCO) coatings, and gas sensors, owing to its excellent electrical conductivity [2], robust chemical adsorption capacity, and high thermal stability [3]. However, SnO_2_ is typically synthesized via low-temperature processes [4], which, in combination with its small grain size, often results in the formation of abundant surface structural defects such as dangling bonds and under-coordinated atoms [5]. These surface defects have been shown to enhance non-radiative recombination at the interfaces, thereby reducing carrier lifetime and impeding efficient charge transport pathways [6]. This, in turn, has been demonstrated to limit the stability and overall performance of SnO_2_-based devices to some extent [7]. To alleviate the adverse impact of surface defects on SnO_2_ performance, a variety of surface passivation strategies have been developed in recent years. These include heteroatom doping [8] and interfacial modification approaches [9]. By modulating the surface chemical environment or the electronic structure of defects [10], these strategies have been shown to effectively reduce defect state densities [11] and suppress non-radiative recombination processes [12], thereby improving carrier dynamics and enhancing device stability [13]. However, conventional doping techniques frequently encounter challenges, including poor selectivity, processing steps, and limited compatibility with underlying substrates, which hinders their practical applicability [14]. Consequently, the development of efficient, controllable, and adaptable passivation strategies has emerged as a primary research focus in the regulation of surface defects in SnO_2_.

In recent years, molecular passivation has emerged as a promising approach for surface defect control and has attracted considerable attention [15]. This strategy is predicated on the coordination interactions between molecules and surface defect sites [16], thereby enabling precise modulation of the local electronic structure and effective passivation of surface defect states [17]. In comparison with conventional doping methodologies, molecular passivation demonstrated superior selectivity and tunability, enabling targeted interactions with specific defect sites and regulation of their properties [18]. Furthermore, the structural diversity and functional tunability of molecular complexes endow them with strong environmental adaptability, providing new insights and pathways for improving the performance of SnO_2_-based materials. This includes organic molecules, inorganic salts, polymer modifiers, and organic–inorganic hybrid materials, which can work synergistically to improve the stability of the SnO_2_ interface. Yuan et al. [19] introduced thiophenol-based ligands (TP-ligands) bearing SH groups and π-conjugated structures into CsPbI_3_ perovskite precursors, leading to the formation of stable CsPbI_3_ films with reduced trap densities and enhanced carrier mobilities. Wang et al. [20] utilized the incorporation of cyano-substituted π-conjugated molecules into perovskite films, a strategy that effectively addressed both surface and grain boundary defects. This approach led to a substantial enhancement in device performance and operational stability. Koseki et al. [21] employed organic molecules with extended π-conjugated frameworks to inhibit molecular desorption, optimize interfacial energy level alignment, and simultaneously improve defect passivation and hole transport in perovskite solar cells. Consequently, the efficiency of the device increased from 22.7% to 24.6%, accompanied by a substantial enhancement in device stability.

This study aims to explore the potential application of molecular complexes in defect passivation on the SnO_2_ (110) surface. To elucidate the underlying mechanism, first-principles calculations were employed to systematically investigate the interactions between the 18-crown-6–potassium complex (18C6-K^+^) and six typical surface defects of SnO_2_ (110), namely Sn_i_ + Sn_O_, O_i_ + O_Sn_, V_O_ + Sn_i_, V_Sn_ + Sn_O_, V_Sn_ + Sn_i_, and Sn_i_. Particular attention was paid to the regulation of adsorption stability, defect formation energies, and electronic effective masses, combined with analyses of band structures, density of states, charge density differences, and Bader charge results, to comprehensively clarify the defect passivation mechanism of 18C6-K^+^. In Section 2, the computational models, parameter settings, and theoretical methods were detailed; In Section 3, we systematically analyzed the adsorption characteristics of 18C6-K^+^ on the SnO_2_ (110) surface, the variations in defect formation energies and electronic effective masses, and revealed its defect passivation mechanism through analyses of band structures, density of states, charge density differences, and Bader charge results. And in Section 4, the main conclusions and highlights the potential application value of the 18C6-K^+^ passivation strategy in enhancing the performance of SnO_2_-based optoelectronic devices were made. This study provides a more complete theoretical foundation and technical guidance for optimizing the performance of SnO_2_-based optoelectronic devices.

## 2. Results and Discussion

### 2.1. Atomic Structures of the 18C6-K^+^ and SnO_2_

**Figure 1:**
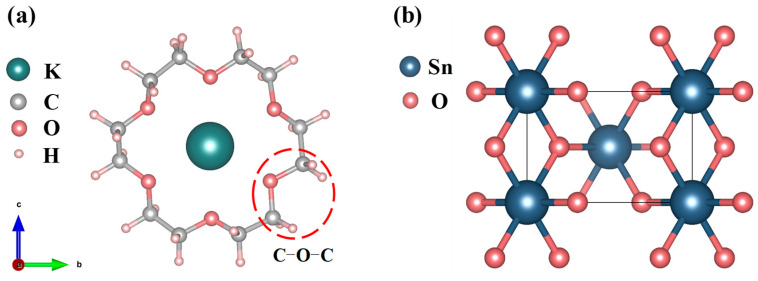
(**a**) Crystal structure of 18C6-K^+^; (**b**) crystal structure of SnO_2_.

**Figure 2:**
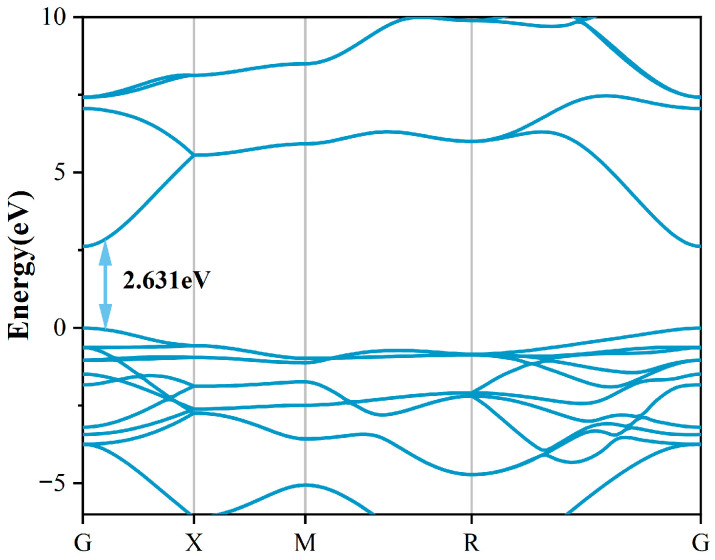
Band structure of pristine SnO_2_.

Figure 1a presents the crystallographic configuration of the 18C6-K^+^ molecular complex. The complex under consideration consists of a crown ether (18C6) composed of 12 saturated carbon atoms and 6 oxygen atoms, forming a ring that encapsulates a K^+^ ion. The potassium ion is located at the geometric center of the ring and forms a stable structure through coordination interactions with the inner-ring oxygen atoms. The selection of 18C6-K^+^ as the passivation molecule is primarily based on its structural advantages, charge regulation capability, and novelty in the field of SnO_2_ defect passivation. To highlight its uniqueness, a comparison with representative passivation molecules for SnO_2_ reported in recent years, along with 18C6-K^+^, is summarized (as shown in Table 1). As depicted in Figure 1b, pristine SnO_2_ adopts a rutile-type crystal structure belonging to the tetragonal space group P42/mnm [22], characterized by typical tetragonal symmetry [23]. Within this lattice, each Sn atom is octahedrally coordinated by six oxygen atoms, while each O atom forms bonds with three Sn atoms, collectively forming a stable three-dimensional crystal framework. Following the structural optimization of the SnO_2_ crystal utilizing the GGA + U method, the obtained lattice parameters were determined to be a = b = 4.689 Å and c = 3.131 Å. These results closely match the experimental lattice constants of a = b = 4.737 Å and c = 3.185 Å [24], confirming the accuracy and reliability of the computational method in describing geometric structures. Additionally, the calculated bandgap is 2.631 eV (as shown in Figure 2), representing a significant enhancement compared to the conventional PBE result of 0.64 eV and closer to the experimental benchmark of 3.6 eV [25]. The underestimation of the SnO_2_ bandgap mainly arises from the systematic errors of the PBE functional in wide-bandgap oxides, including the absence of derivative discontinuity and the incomplete cancelation of electron self-interaction, which result in an artificially lowered conduction band energy. This error has a greater impact on unoccupied states but only a minor effect on occupied states; therefore, the structural parameters, relative energetics, and defect thermodynamics remain reliable. This study focuses on the relative trends of defect formation energies, defect state distributions, and electronic effective masses before and after passivation, which are insensitive to the choice of U values and thus reliable. Compared with PBE, the GGA + U method more reasonably corrects the correlation between Sn-4d and O-2p states, significantly improving the accuracy of describing the electronic structure of SnO_2_, making the results closer to experimental observations and more physically rigorous.

### 2.2. Adsorption Behavior of 18C6-K^+^ on the SnO_2_ (110) Surface

#### 2.2.1. Stability of the Adsorption System

**Figure 3:**
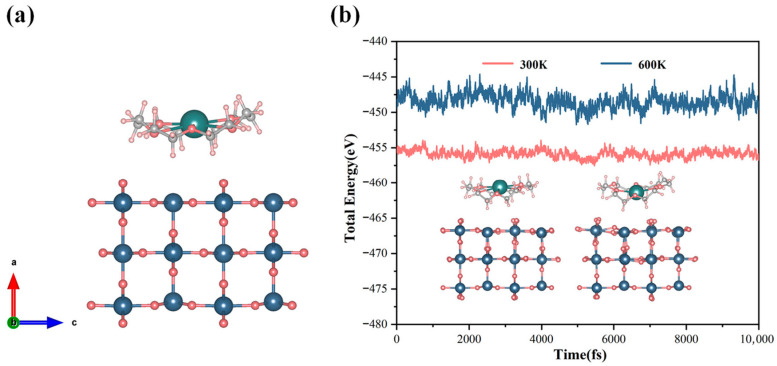
(**a**) Optimized adsorption model of 18C6-K^+^ on the SnO_2_ (110) surface; (**b**) AIMD simulations of the adsorption system performed at 300 K and 600 K within the NVT ensemble framework.

Extensive experimental and theoretical studies have demonstrated that the (110) surface, owing to its lowest surface formation energy, is the most thermodynamically stable and most commonly exposed facet [31]. In SnO_2_ films prepared by solution processing, ALD, or sputtering, the (110) plane is consistently the dominant exposed surface, and the (110) diffraction peak remains the most prominent in XRD θ−2θ scans for films with a thickness ≤ 100 nm [32]. Consequently, the SnO_2_ (110) surface was selected in this study, and a slab with 3 × 2 × 1 supercell was constructed for surface structure. To eliminate artificial interactions between periodic images along the surface normal, a vacuum region of 15 Å was introduced. During the process of structural optimization, the bottom atomic layer was constrained, while all other atoms were fully relaxed to obtain a realistic and stable surface structure. Subsequent to surface relaxation, the 18C6-K^+^ was positioned on the optimized SnO_2_ (110) surface, and additional relaxation was executed to attain a stable adsorption configuration (see Figure 3a). The thermal stability of the adsorption configuration was assessed via ab initio molecular dynamics (AIMD) simulations conducted under the canonical (NVT) ensemble. The time step was set to one fs to ensure the precise capture of phonon vibrations, with the total simulation time fixed at 10,000 fs. As demonstrated in Figure 3b, the total energy of the system fluctuated marginally without any substantial energy drift at both 300 K and 600 K. Furthermore, the overall geometry manifested only negligible distortions, with no occurrence of bond breakage or molecular desorption. These results indicate that the constructed 18C6-K^+^/SnO_2_ (110) adsorption model exhibits excellent thermodynamic stability across a range of temperatures, suggesting that it can maintain structural integrity under practical working conditions and holds great potential for real-world applications.

Furthermore, to further verify the stability of the adsorption system, the adsorption energy of the 18C6-K^+^ on the SnO_2_ (110) surface was determined using the following equation:

(1) $$E_{ads}=E_{18C6-K^{+}/surface}-E_{surface}-E_{18C6-K^{+}}$$

where $E_{18C6-K^{+}/surface}$ represents the total energy of 18C6-K^+^ adsorbed on the SnO_2_ (110) surface, $E_{surface}$ refers to the energy of the pristine SnO_2_ (110) surface, and $E_{18C6-K^{+}}$ corresponds to the energy of the isolated 18C6-K^+^ in vacuum. The detailed energy values are listed in Table 2. The calculated adsorption energy of 18C6-K^+^ on the SnO_2_ (110) surface is −3.884 eV, indicating a strong thermodynamic favorability and robust binding interaction at the interface. This result further verifies that 18C6-K^+^ can form a thermodynamically stable adsorption configuration on the SnO_2_ (110) surface.

#### 2.2.2. Electronic Properties of Adsorption System

**Figure 4:**
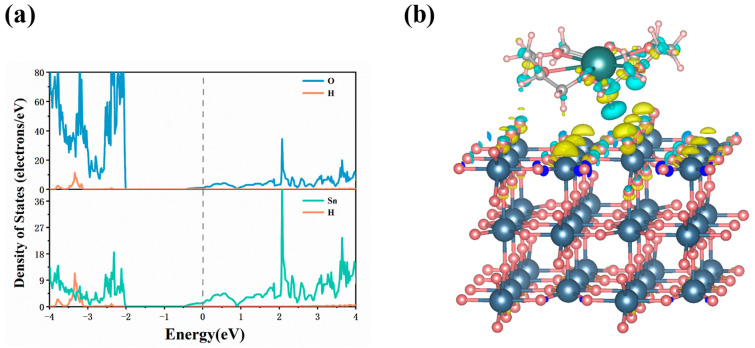
(**a**) PDOS of the 18C6-K^+^ adsorbed on the SnO_2_ (110) surface; (**b**) CDD map of the adsorption system. The isosurface was set at 0.001 bohr^−3^, where the cyan and yellow regions represent charge depletion and accumulation, respectively.

To gain deeper insights into the interaction between 18C6-K^+^ and the SnO_2_ (110) surface, a systematic analysis of the projected density of states (PDOS) and charge density difference (CDD) of the adsorption system was performed. As illustrated in Figure 4a, the adsorption process leads to a noticeable electronic overlap between the Sn and O atoms on the SnO_2_ (110) surface and the hydrogen atoms of 18C6-K^+^, indicating a strong electronic interaction between 18C6-K^+^ and the substrate. Furthermore, as illustrated in Figure 4b, there is a depletion of charge around the H, C, and O atoms of 18C6-K^+^, accompanied by evident charge accumulation on the Sn and O atoms of the SnO_2_ (110) surface. This redistribution of charge suggests electron transfer from the ether linkages (C-O-C) in 18C6-K^+^ to the Sn and O atoms on the surface. This transfer results in the formation of stable Sn–ether and O–ether interactions, which, in turn, enhance the adsorption stability of the molecule on the surface.

### 2.3. Passivation Effect of 18C6-K^+^ on Surface Defects of SnO_2_

**Figure 5:**
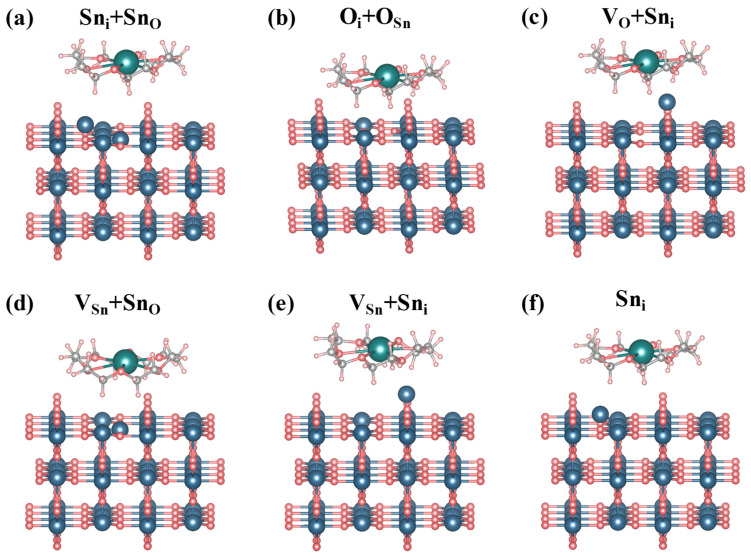
Adsorption model of 18C6-K^+^ on defective SnO_2_ (110) surface. Here, “V” and “i” stand for vacancy and interstitial, respectively.

In this study, defective SnO_2_ (110) surface models were constructed (Figure 5), and 18C6-K^+^ was introduced onto the defect regions to investigate its passivation effect on surface defects. Six representative surface defects, Sn_i_ + Sn_O_, O_i_ + O_Sn_, V_O_ + Sn_i_, V_Sn_ + Sn_O_, V_Sn_ + Sn_i_, and Sn_i_ were selected for passivation due to their prevalence and significant impact on material properties as intrinsic or complex defect types. These defects introduce surface states that affect the electrical and optical performance of SnO_2_. From an energetic perspective, isolated point defects (such as single oxygen vacancies) typically exhibit high formation energies and chemical reactivity. The system tends to reduce its overall free energy—and thereby achieve greater stability—through vacancy aggregation, complexation with other defects, or by inducing surface reconstructions. So, this study focused on defect complexes, namely V_O_ + Sn_i_, instead of single V_O_. An initial evaluation was performed on the adsorption energies of 18C6-K^+^ across different defect sites on SnO_2_ surface. The definition of adsorption energy is based on the total energy difference of the supercell rather than the energy difference of a single atom; therefore, its value usually falls within the energy range of forming one or two chemical bonds. Under different defect environments, the relative magnitude of the adsorption energy can effectively reflect the differences and trends in binding strength and structural stability. More negative values reflect stronger interactions with defect sites and enhanced thermodynamic stability of the corresponding adsorption configurations. The detailed energy values are listed in Table 2. As demonstrated in Table 3, All adsorption configurations exhibit negative adsorption energies, thereby substantiating the thermodynamic favorability of the interactions. Among them, the O_i_ + O_Sn_ defect site is the most favorable adsorption site, with an energy of −6.117 eV, suggesting the strongest interaction with 18C6-K^+^.

#### 2.3.1. Defect Formation Energy

To assess the defect passivation effect induced by 18C6-K^+^ on SnO_2_, the formation energies of six representative surface defects of SnO_2_ (110), i.e., Sn_i_ + Sn_O_, O_i_ + O_Sn_, V_O_ + Sn_i_, V_Sn_ + Sn_O_, V_Sn_ + Sn_i_, and Sn_i_, were calculated before and after 18C6-K^+^ adsorption under both Sn_rich_ and O_rich_ growth conditions. The total energies of the related systems are summarized in Table 4, while the calculated defect formation energies are listed in Table 5. The defect formation energy (*E_f_*) was determined according to the following expression:

(2) $$E_{f}=E_{defective\;-\;}E_{pristine}+\sum_{i}n_{i}\left(\mu_{i+}E_{i}\right)\;\;\;\;\;\;\;\;\;\;\;\;\;\;\;\;\;\;\;\;\;\;\;\;\;\;\;\;\;\;\;\;$$

where $E_{defective}$ denotes the total energy of the system containing the defect, while $E_{pristine}$ refers to the total energy of the pristine (defect-free) structure. $n_{i}$ represents the number of atoms of species *i* that are removed from (positive $n_{i}$) or added to (negative $n_{i}$) the system, $\mu_{i}$ representing the chemical potential in equilibrium with the growth environment, and $E_{i}$ corresponds to the total energy of element *i* in its standard reference state. Although $E_{defective}$ already includes the total energy of the system after structural and compositional changes, it does not account for the exchange energy with external atomic reservoirs. The $\sum_{i}ni\mu i$ term explicitly incorporates the effect of stoichiometric variation and environmental conditions, thereby ensuring the comparability of supercells with different compositions. In this work, the reference energies were set as follows: $E_{SnO2\;}$= −19.835 (eV), $E_{Sn}\;$= −3.975 (eV), and $E_{O2}\;$= −9.694 (eV). The chemical potentials were determined under the thermodynamic constraint $\mu_{Sn}\;$+ 2$\mu_{O\;}\;$=$\;∆Hf({SnO}_{2})$. By imposing the upper bounds, the Sn-rich and O-rich limiting conditions were defined, and the corresponding chemical potential values were obtained. These values were then substituted into the defect formation energy formula for the final calculations (see Table 5). A lower defect formation energy suggests a higher probability of defect formation under the specified chemical potential conditions.

**Figure 6:**
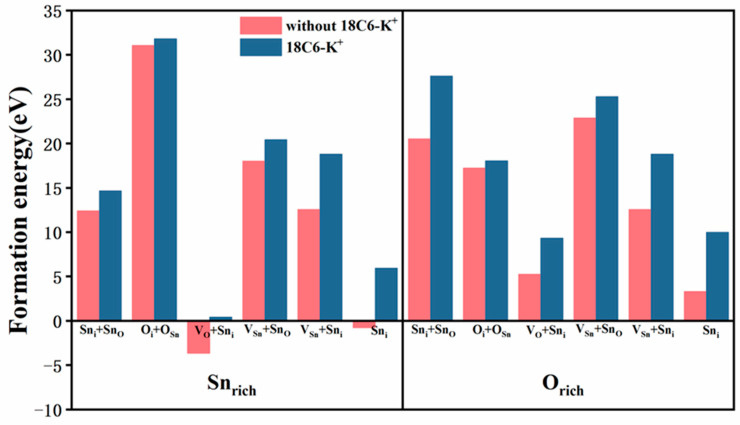
Defect formation energies (*E_f_*) of various defect configurations on the SnO_2_ (110) surface, with and without 18C6-K^+^ adsorption, under Sn_rich_ and O_rich_ growth conditions, respectively.

As demonstrated in Figure 6, the Sn_i_ + Sn_O_, V_O_ + Sn_i_, V_Sn_ + Sn_O_, V_Sn_ + Sn_i_, and Sn_i_ defects manifest lower formation energies under Sn_rich_ condition compared to O_rich_ condition, suggesting that these defects are more prone to form under Sn_rich_ environment. Conversely, the O_i_ + O_Sn_ defect exhibits a lower formation energy under O_rich_ condition. Moreover, following the introduction of the 18C6-K^+^, the formation energies of all defects showed a significant increase under both Sn_rich_ and O_rich_ conditions. These results imply that the interaction between 18C6-K^+^ and defect sites increases the formation energy of defects, thereby effectively inhibiting their generation. These findings suggest that 18C6-K^+^ has the potential to passivate interface defects, which is expected to enhance the structural stability of SnO_2_ materials and improve device performance.

#### 2.3.2. Electron Effective Mass

This study focuses on the primary role of SnO_2_ as an n-type electron transport layer (ETL) in solar cells. In this system, regardless of whether a passivation layer is introduced, minority carrier holes mainly participate through recombination with electrons, and their contribution can generally be neglected. Therefore, to further evaluate the impact of the 18C6-K^+^ on the electronic transport characteristics of defective SnO_2_ (110) surface, the electron effective masses ($m^{*}$) were computed. The corresponding values are presented in Table 6. $m^{*}$ is defined by the following equation:

(3) $$\frac{1}{m^{*}}=\frac{1}{h^{2}}\frac{\partial^{2}E\left(k\right)}{\partial k^{2}}\;\;$$

where *h* denotes Planck’s constant, *k* represents the electron wave-vector (momentum), and *E(k)* stands for the electronic energy as a function the wave vector *k*. The calculations were performed using a Monkhorst–Pack grid, and the electron effective mass was obtained at the conduction band minimum (CBM) at the Γ point, with the specific values determined along the Γ→R direction. The results demonstrate that, following the introduction of the 18C6-K^+^ to various defect sites, there is a consistent reduction in *m ^⁎^* suggesting that the presence of the 18C6-K^+^ could facilitate carrier migration and enhances the overall charge transport capability on the surface. The coordination between K^+^ ions and the 18C6 ligand facilitates electron donation to the SnO_2_ (110) surface, thereby partially filling defect states and mitigating charge carrier localization effects. Concurrently, the 18C6-K^+^ molecule has the capacity to passivate surface defects through either physisorption or the formation of weak chemical bonds, thereby effectively reducing the density of surface states. This passivation has been demonstrated to reduce electron scattering, thereby optimizing transport performance. Furthermore, the incorporation of K^+^ may modulate the surface band structure to a slight extent, enhancing band delocalization and further promoting electron mobility. In summary, the 18C6-K^+^ could effectively reduce the electron effective mass of the SnO_2_ (110) surface through a synergistic combination of charge transfer and band structure modulation. In this work, the reduction in $m^{*}$ is considered only indicative evidence of improved transport trends, rather than direct proof of an absolute increase in mobility. These improvements have been shown to enhance the electron transport properties of SnO_2_ significantly, and they offer a novel interfacial engineering strategy for the development of high-performance electronic devices.

#### 2.3.3. Density of States and Band Structure

**Figure 7:**
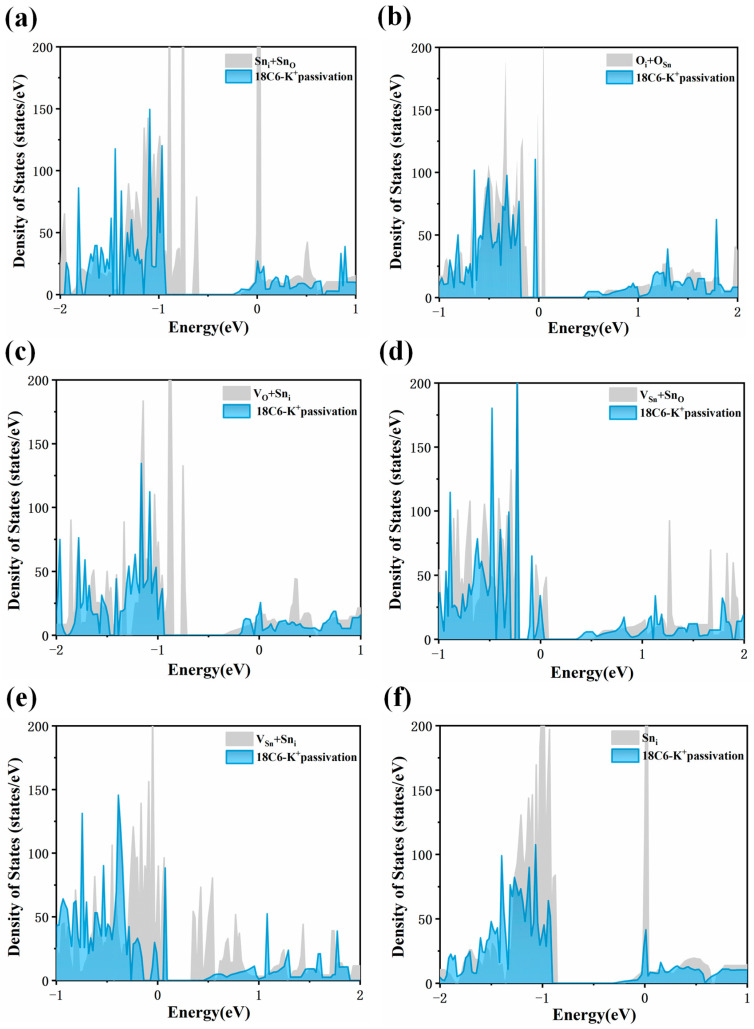
DOS of defective SnO_2_ (110) surface with (**a**) Sn_i_ + Sn_O_, (**b**) O_i_ + O_Sn_, (**c**) V_O_ + Sn_i_, (**d**) V_Sn_ + Sn_O_, (**e**) V_Sn_ + Sn_i_, and (**f**) Sn_i_ before and after 18C6-K^+^ adsorption. The gray areas represent the density of states prior to adsorption, whereas the blue areas indicate the DOS following adsorption.

A systematic evaluation was performed to further investigate the defect healing ability of 18C6-K^+^ on the SnO_2_ (110) surface. Accordingly, total density of states (DOS) analyses was carried out for six representative surface defects before and after adsorption of 18C6-K^+^. As shown in Figure 7a,c, after introducing 18C6 K^+^ to the SnO_2_ (110) surface containing Sn_i_ + Sn_O_ and V_O_ + Sn_i_ defects, the defect-induced electronic states initially located in the bandgap undergo a complete disappearance. As illustrated in Figure 7b,d, for the O_i_ + O_Sn_ and V_Sn_ + Sn_O_ defects, the incorporation of the 18C6-K^+^ results in a significant reduction in the peaks of defect-related states within the bandgap, close to the valence band maximum (VBM), indicating that the 18C6-K^+^ displays a moderate healing effect on these defects, thereby mitigating their impact on the electronic structure to a certain extent, though not completely eliminating the associated defect states. A subsequent examination of Figure 7e,f indicates that for surfaces containing V_Sn_ + Sn_i_ and Sn_i_ defects, the defect-related peaks near the CBM are significantly reduced or nearly eliminated upon adsorption of the 18C6-K^+^, suggesting that the adsorption of the 18C6-K^+^ effectively repairs these defects and restores the electronic structure of the SnO_2_ (110) surface, particularly in the conduction band region, thereby significantly enhancing the material’s electron transport properties. In summary, the adsorption of 18C6-K^+^ on the SnO_2_ (110) surface can eliminate the defect states of all six types of surface defects.

**Figure 8:**
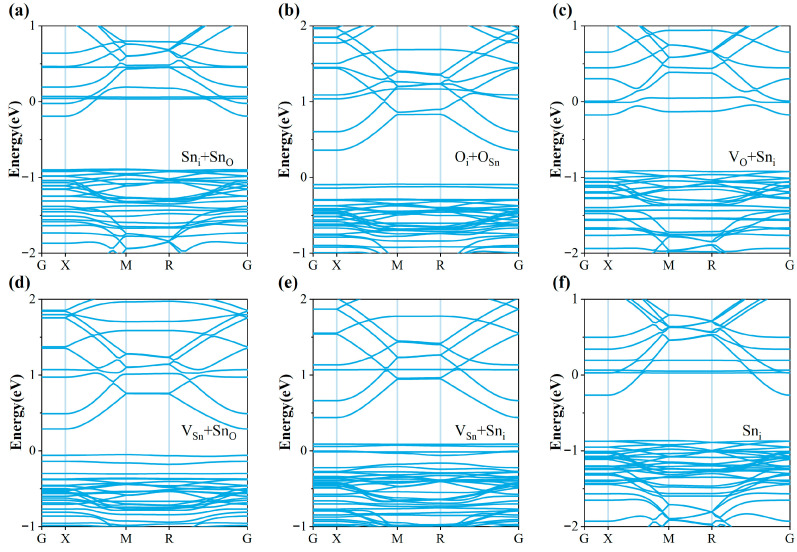
Band structures of SnO_2_ (110) surfaces with (**a**) Sn_i_ + Sn_O_, (**b**) O_i_ + O_Sn_, (**c**) V_O_ + Sn_i_, (**d**) V_Sn_ + Sn_O_, (**e**) V_Sn_ + Sn_i_, (**f**) Sn_i_ defects after 18C6-K^+^ adsorption.

In addition, we calculated the band structure of the system after 18C6-K^+^ adsorption (Figure 8) to further verify the accuracy and reliability of the DOS results. The results show that the band curvature is highly consistent with the density of states distribution, and the defect states within the bandgap are significantly suppressed or completely eliminated after passivation. This not only agrees well with the DOS analysis, but also demonstrates that 18C6-K^+^ effectively eliminates defect-induced deep levels, restores a band structure close to that of ideal SnO_2_, and reduces the electron effective mass, thereby facilitating improved carrier transport. Therefore, with the dual verification of DOS and band structures, it can be clearly confirmed that the adsorption of 18C6-K^+^ effectively repairs band distortions caused by multiple defects and significantly improves the electronic structure and transport properties of the SnO_2_ (110) surface. In particular, strong passivation effects were observed for the Sn_i_ + Sn_O_, V_O_ + Sn_i_, V_Sn_ + Sn_i_, and Sn_i_ defects, thereby effectively enhancing the electronic structure and interfacial characteristics.

#### 2.3.4. Charge Density Difference

**Figure 9:**
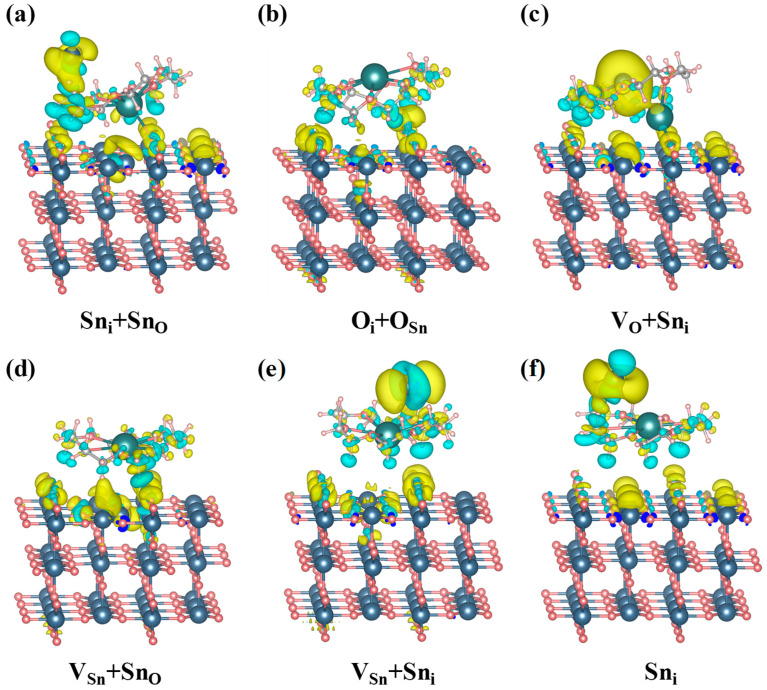
CDD maps of the 18C6-K^+^ adsorbed on defective SnO_2_ (110) surface with (**a**) Sn_i_ + Sn_O_, (**b**) O_i_ + O_Sn_, (**c**) V_O_ + Sn_i_, (**d**) V_Sn_ + Sn_O_, (**e**) V_Sn_ + Sn_i_, and (**f**) Sn_i_. The isosurface was set at 0.001 bohr^−3^, where the cyan and yellow regions represent charge depletion and accumulation, respectively.

To better understand the 18C6-K^+^ adsorption mechanism and charge transfer on SnO_2_ (110), charge density difference (CDD) analyses were performed. The CDD is computed using the following equation:

(4) $$\Delta \rho =\rho_{18C6\;-\;K^{+}/surface}{\;-\;\rho }_{surface}\;-\;\rho_{18C6\;-\;K^{+}}\;$$

where $\rho_{18C6-K^{+}/surface\;\;,\;\;\;}\rho_{surface}$ and $\rho_{18C6-K^{+}}$ represent the charge densities of the 18C6-K^+^ adsorbed on the SnO_2_ (110) surface, the bare SnO_2_ (110) surface, and the isolated 18C6-K^+^ molecule, respectively. As shown in Figure 9, the 18C6-K^+^ molecule exhibits significant charge transfer behavior across all investigated defect configurations, indicating its strong capability for defect passivation. Specifically, when 18C6-K^+^ is adsorbed at the Sn_i_ + Sn_O_ defect site as shown in Figure 9a, pronounced charge depletion occurs around the hydrogen, carbon, and oxygen atoms of the molecule, suggesting that electrons are transferred from the molecule to the SnO_2_ surface. Simultaneously, charge accumulation is observed around the adjacent tin atoms and a portion of the oxygen atoms near the defect region, indicating the formation of interfacial interactions dominated by tin ether coordination, complemented by oxygen ether coordination. This electron redistribution enhances the coupling between the molecule and the defect site, thereby enabling effective passivation of the Sn_i_ + Sn_O_ defect. In the V_O_ + Sn_i_ (Figure 9c), V_Sn_ + Sn_O_ (Figure 9d), and Sn_i_ (Figure 9f) defect configurations, comparable charge depletion occurs around the hydrogen, carbon, and oxygen atoms of the 18C6-K^+^ molecule, while charge accumulation is primarily located near the tin atoms on the SnO_2_ surface. These findings suggest that electron transfer predominantly occurs from the molecule’s ether groups (C–O–C) to the surface tin atoms, facilitating the formation of tin–ether bonds which effectively passivate surface defects and enhance interfacial stability. For the O_i_ + O_Sn_ defect site shown in Figure 9b, charge depletion remains concentrated within the 18C6-K^+^ molecule, whereas charge accumulation predominantly occurs around the surface oxygen atoms of SnO_2_. This suggests that electrons are transferred from the ether groups to surface oxygen atoms, forming oxygen ether interactions that contribute to the repair of oxygen related defects. In the V_Sn_ + Sn_i_ defect configuration shown in Figure 9e, the hydrogen, carbon, and oxygen atoms of 18C6-K^+^ continue to show charge depletion, while charge accumulation simultaneously appears around both tin and oxygen atoms near the defect site. This observation reflects the complexity of this particular defect environment, where the coexistence of tin ether and oxygen ether interactions enables synergistic passivation of the complex defect structure. In summary, the 18C6-K^+^ donates electrons from its ether (C-O-C) groups to Sn and O atoms at the defect sites on the SnO_2_ (110) surface, forming Sn-ether and O-ether interactions. These interactions enable effective passivation of diverse surface defects and substantially enhance the electronic structure of the SnO_2_ (110) surface.

To quantitatively evaluate the charge transfer results revealed by CDD, Bader charge analysis was performed (see Table 7). The results showed that the H, C, and O atoms in the 18C6-K^+^ molecule generally lose electrons, while the Sn or O atoms at the defect sites on the surface gain electrons, which is highly consistent with the charge density redistribution trend observed in CDD. Specifically, in Figure 9a, the C and O atoms lose approximately 0.49 e and 1.14 e, respectively, while the Sn and O atoms on the SnO_2_ (110) surface gain about 2.5 e and 1.29 e, respectively. In Figure 9c, the C and O atoms lose about 0.52 e and 1.09 e, whereas the Sn and surface O atoms gain about 1.69 e and 1.17 e, respectively. Other defect models exhibit the same electron transfer pattern. In summary, the Bader charge analysis and CDD results mutually corroborate each other, quantitatively confirming the mechanism of electron transfer from 18C6-K^+^ to the defect regions of SnO_2_.

Meanwhile, 18C6-K^+^ effectively suppresses deep defect states within the bandgap through coordination interactions and charge compensation. Such defect states typically act as recombination centers that shorten carrier lifetimes. Their suppression implies reduced recombination pathways, thereby potentially extending carrier lifetimes. The density of states and charge density difference results support the reasonable inference that 18C6-K^+^ contributes to the dual improvement of both effective mass and carrier lifetime.

## 3. Computational Methods

In this study, structural optimizations, total energy computations, and evaluations of interfacial characteristics were conducted through first-principles methods based on density functional theory (DFT) [33], utilizing the Vienna Ab initio Simulation Package (VASP) (Version 6.4.1) [34]. The projector augmented wave (PAW) method was employed to model the electron–ion interactions [35], the exchange and correlation contributions were addressed by the Perdew–Burke–Ernzerhof (PBE) functional under the generalized gradient approximation (GGA) framework [36]. However, the GGA method often falls short in accurately describing the properties of strongly correlated systems, especially those involving highly localized d or f electrons [37]. To overcome this shortcoming, the GGA + U approach, incorporating the Hubbard U correction, was utilized in this study to better capture the behavior of localized electronic states [38]. While the valence electrons of Sn atoms in SnO_2_ are mainly derived from s and p orbitals, the 4d orbitals also play a significant role in shaping the material’s electronic structure [39]. To better account for the localized behavior of these 4d states and their effects on the band structure, optical response, and charge transport, the generalized gradient approximation with Hubbard U correction (GGA + U) was applied in this work. The Hubbard U values were obtained via the linear response approach, resulting in 4.17 eV for Sn 4d states and 8.4 eV for O 2p states. Consequently, on-site Coulomb interaction parameters of U_(Sn,d)_ = 4.17 eV and U_(O,p)_ = 8.4 eV were assigned to the 4d^10^5s^2^5p^2^ electron configuration of Sn and the 2s^2^2p^4^ configuration of O, respectively. A 4 × 4 × 1 k-point mesh generated by the Monkhorst–Pack scheme was used for Brillouin zone sampling, and the plane-wave basis set was truncated at an energy cut-off of 500 eV. Convergence thresholds were set as 0.02 eV/nm for atomic forces, 1 × 10^−5^ eV/atom for the total energy in self-consistent field (SCF) calculations, and 0.02 GPa for the residual stress.

## 4. Conclusions

This study employed first-principles calculations to systematically explore the defect passivation mechanisms of 18C6-K^+^ at six representative defect sites (i.e., Sn_i_ + Sn_O_, O_i_ + O_Sn_, V_O_ + Sn_i_, V_Sn_ + Sn_O_, V_Sn_ + Sn_i_, and Sn_i_) of the SnO_2_ (110) surface. These intrinsic and complex defects are commonly present and significantly influence the material’s performance by creating surface defect states that alter the electrical and optical behavior of SnO_2_. The results confirmed that the 18C6-K^+^ molecule can stably adsorb at all six defect sites, exhibiting favorable thermodynamic stability. Moreover, inducing 18C6-K^+^ on SnO_2_ (110) surface could increase defect formation energies, indicating a strong potential for suppressing defect generation. Furthermore, the presence of 18C6-K^+^ has been shown to reduce the electron effective mass of defective systems, thereby enhancing electron mobility and interfacial charge transport efficiency. DOS analyses revealed that 18C6-K^+^ exhibits strong passivation effects on Sn_i_ + Sn_O_, V_O_ + Sn_i_, V_Sn_ + Sn_i_, and Sn_i_ defects, while also partially mitigating the electronic disturbances caused by O_i_ + O_Sn_ and V_Sn_ + Sn_O_ defects. Consequently, this results in improved structural stability and electronic performance of SnO_2_. The corresponding band structure results are highly consistent with the DOS analysis, further confirming its effective defect-healing capability. CDD analysis substantiated that the 18C6-K^+^ molecule forms Sn-ether and O-ether interactions via its ether bonds (C-O-C) with surface Sn and O atoms, effectively passivating defect states and suppressing SnO_2_ decomposition. The Bader charge results indicate that the H, C, and O atoms in the 18C6-K^+^ molecule generally lose electrons, while the Sn or O atoms at the surface defect sites gain electrons. The Bader charge analysis is consistent with the CDD results, quantitatively confirming the mechanism of electron transfer from 18C6-K^+^ to the SnO_2_ defect regions. In summary, 18C6-K^+^ functions as an efficient molecular passivator, providing a promising interfacial engineering strategy and theoretical basis for enhancing the performance of SnO_2_-based materials and devices.

## Figures and Tables

**Table 1:** Comparison of passivation mechanisms in passivation molecules for SnO_2_.

Passivation Molecule	Mechanism	Reference
O-phospho-L-serine monolithium salt	Phosphate/carboxyl groups coordinate with SnO_2_; improved conductivity and stability	[26]
Ectoine	Carboxyl groups bind with Sn^4+^/oxygen vacancies; imine groups coordinate with Pb^2+^, enhancing stability	[27]
Thiourea	-NH_2_ and S atoms with lone pairs interact with Sn^4+^/oxygen vacancy, improving efficiency	[28]
Phosphorylcholine chloride	Passivation of SnO_2_ and interface defects; PCE improvement	[10]
(2-aminoethyl) phosphonic acid	Phosphonic acid groups coordinate with SnO_2_, suppressed recombination; PCE increased from 19.65% to 28.36%	[29]
Sulfamate sodium 4-aminoazobenzene-4′-sulfonat	Passivation of SnO_2_ anion/cation vacancies; improved interfacial contact and stability	[30]
18C6-K^+^	Sn–ether/O–ether coordination; K^+^ charge transfer; suppressed complex defects, improved transport	This work

**Table 2:** Numerical values of energy terms corresponding to adsorption energy (eV).

Defect Type	$E_{18C6-K^{+}/surface}$ (eV)	$E_{surface}$ (eV)	$E_{18C6-K^{+}}$ (eV)
pristine	−460.350	−257.444	−199.022
Sn_i_ + Sn_O_	−440.830	−236.897	−199.328
O_i_ + O_Sn_	−438.231	−232.528	−199.586
Vo + Sn_i_	−455.074	−252.596	−199.704
V_Sn_ + Sn_O_	−435.060	−231.027	−199.596
V_O_ + Sn_i_	−441.539	−241.095	−199.856
Sn_i_	−454.403	−251.881	−199.023

**Table 3:** Adsorption energies (in eV) of 18C6-K^+^ on defective SnO_2_ (110) surface.

Defect Site	Adsorption Energy (eV)
Sn_i_ + Sn_O_	−4.605
O_i_ + O_Sn_	−6.117
V_O_ + Sn_i_	−2.774
V_Sn_ + Sn_O_	−4.437
V_O_ + Sn_i_	−0.588
Sn_i_	−3.499

**Table 4:** Total energies (eV) of the pristine and various defective SnO_2_ (110) surface with 18C6-K^+^ adsorption.

Defect Type	Energy (eV)
Pristine structure	−460.350
Sn_i_ + Sn_O_	−440.830
O_i_ + O_Sn_	−438.231
V_O_ + Sn_i_	−455.074
V_Sn_ + Sn_O_	−435.060
V_Sn_ + Sn_i_	−441.539
Sn_i_	−454.403

**Table 5:** Defect formation energy (*E_f_*) values for various defect types on the SnO_2_ (110) surface, with/without 18C6-K^+^ adsorption, under Sn_rich_ and O_rich_ growth conditions, respectively.

Defect	Growth Conditions	*E_f_* (eV)	
		Without 18C6-K^+^	With18C6-K^+^
Sn_i_ + Sn_O_	Sn_rich_	12.425	14.672
	O_rich_	20.528	27.622
O_i_ + O_Sn_	Sn_rich_	31.058	31.813
	O_rich_	17.252	18.067
V_O_ + Sn_i_	Sn_rich_	−3.646	0.428
	O_rich_	5.252	9.327
V_Sn_ + Sn_O_	Sn_rich_	18.029	20.443
	O_rich_	22.876	25.293
V_Sn_ + Sn_i_	Sn_rich_	12.558	18.821
	O_rich_	12.557	18.821
Sn_i_	Sn_rich_	−0.745	5.947
	O_rich_	3.306	9.998

**Table 6:** Electron effective mass ($m^{*}$) of defective SnO_2_ (110) surface with/without 18C6-K^+^ adsorption.

Defect	Electron Effective Mass	
	Without 18C6-K^+^	With18C6-K^+^
V_O_ + Sn_i_	15.240	0.009
V_Sn_ + Sn_i_	10.668	0.005
O_i_ + O_Sn_	42.671	0.011
Sn_i_	15.240	0.882
V_Sn_ + Sn_O_	8.890	0.004
Sn_i_ + Sn_O_	42.671	0.005

**Table 7:** Bader charge analysis of the adsorption systems (“+” indicates electron gain; “−” indicates electron loss; “surface” refers to atoms of the SnO_2_ (110) surface).

Adsorption Model	Atom	Bader Charge(e)	Adsorption Model	Atom	Bader Charge(e)
Sn_i_ + Sn_O_	H	−0.15	O_i_ + O_Sn_	H	−0.05
	C	−0.49		C	−0.34
	O	−1.14		O	−0.14
	K	−0.9		K	−0.91
	Sn	2.5		Sn	2.34
	O(surface)	1.29		O(surface)	1.23
V_O_ + Sn_i_	H	−0.11	V_Sn_ + Sn_O_	H	−0.19
	C	−0.52		C	−0.43
	O	−1.09		O	−0.95
	K	−0.83		K	−0.9
	Sn	1.69		Sn	2.49
	O(surface)	1.17		O(surface)	1.28
V_Sn_ + Sn_i_	H	−0.17	Sn_i_	H	−0.06
	C	−0.53		C	−0.4
	O	−0.9		O	−1.14
	K	−0.88		K	−0.89
	Sn	2.51		Sn	−2.29
	O(surface)	1.28		O(surface)	1.21

## Data Availability

Data will be made available on request.
